# Preventive Effect of the Herbal Preparation, HemoHIM, on Cisplatin-Induced Immune Suppression

**DOI:** 10.1155/2019/3494806

**Published:** 2019-03-20

**Authors:** Seul-Ki Kim, Da-Ae Kwon, Hak Sung Lee, Hyun Kyu Kim, Won-Ki Kim

**Affiliations:** ^1^Department of Neuroscience, Korea University College of Medicine, 145, Anam-ro, Seongbuk-gu, Seoul 02841, Republic of Korea; ^2^Food Science R&D Center, Kolmar BNH Co., LTD, 22-15, Sandan-gil, Jeonui-myeon, Sejong-si 30003, Republic of Korea

## Abstract

We determined the functional effect of the herbal preparation, HemoHIM, on the immune system, by examining the immunomodulatory activities of HemoHIM using immunocompromised mice. In this study, to examine the effect on the restoration of immune cells and balance in the immune system, we utilized a cisplatin-induced immunosuppression mouse model. Mice were injected intraperitoneally with cisplatin, an immunosuppressive anticancer, and then received oral doses of 100, 250, and 500 mg/kg of HemoHIM for 14 days. The HemoHIM prevented the cisplatin-induced loss of body and organ weight. In terms of innate immunity, natural killer (NK) cell activity and phagocytosis increased in the HemoHIM group compared to the cisplatin control group. The HemoHIM group also showed a significantly higher expression of Th1-mediated cytokines (interferon gamma (IFN-*γ*), interleukin-2 (IL-2), and tumor necrosis factor alpha (TNF-*α*)) and inhibited the production of Th2-mediated cytokine interleukin-4 (IL-4) compared to cisplatin control group. These findings indicate that HemoHIM enhances immune activity by modulating immune cell activity and cytokine secretion in immune-suppressed mice.

## 1. Introduction

Rapid economic growth and advances in medical technology in recent years have prolonged the human lifespan. However, the extended lifespan causes exposure to adult diseases and immune-related diseases such as autoimmune diseases (rheumatoid arthritis and systemic sclerosis) [1], human immunodeficiency, and virus infection [2, 3]. Many researchers studied solving this problem by researching natural products that can regulate the immune system. Pyo et al. [4] concluded that* Phellinus linteus *was able to increase humoral immunity and inhibit immunotoxicity induced by cyclophosphamide and Kim et al. [5] demonstrated that* Cordyceps militaris* enhanced the immune function by promoting Th1 cytokine production and immune cell proliferation. Due to the demand of therapeutic agents that can modulate cell homeostasis and immunodeficiency, there is an increase in studies on the immunomodulatory effects of natural substances [6]. In in vitro and in vivo experiments, the polysaccharides isolated from natural materials can enhance the immune system. Moreover, they present relatively low toxicity and few side effects compared to synthetic drugs. Therefore, natural-substance-derived components are promising candidates for immune response modifiers [7, 8].

HemoHIM is a hot water extract with a polysaccharide fraction of three immunomodulatory herbs,* Angelica gigas *Nakai,* Cnidium officinale *Makino, and* Paeonia japonica *Miyabe [9, 10]. The major chemical constituents of HemoHIM are chlorogenic acid, paeoniflorin, and nodakenin [11]. HemoHIM inhibits various activities of human mast cells [11] and reduces 2,4,6-trinitrobenzene sulfonic acid (TNBS)-induced inflammatory responses in colitis in rats [12]. In addition, HemoHIM has antitumor effects, during chemotherapy and radiotherapy [11, 13, 14]. In particular, HemoHIM affects the differentiation of dendritic cells and regulates immune cells such as spleen and macrophages [15].

Cisplatin, a platinum-based antitumor drug, is the most commonly used compound in medicine. Cisplatin attacks growing tumor cells by inducing apoptosis via the inhibition of DNA synthesis [16]. Recently, many reports have focused on the immunomodulatory properties of cisplatin. Cisplatin induces immunomodulatory effects through inhibiting the proliferation of peripheral lymphocytes in response to allergenic cells or mitogens, the growth of B and T cells, and interruption of chemotaxis among monocytes [17, 18].

No prior reports on HemoHIM have shown an immunomodulatory effect on cisplatin-induced immunosuppression. In the present study, we aim to evaluate the effect of the immunity enhancement of HemoHIM. We investigated changes in cisplatin-induced immunosuppressed mice model by investigating the macrophage phagocytosis, cytokines production, natural killer (NK) cell activity, and splenocyte proliferation.

## 2. Materials and Methods

### 2.1. Animals

Nine-week-old male C57BL/6 mice were purchased from Orient Bio Inc. (Seongnam, Republic of Korea) and housed in ventilated cages under specific pathogen-free (SPF) conditions at Genia Institution (Seongnam, Republic of Korea). All animal procedures were approved by the Institutional Animal Care and Use Committee (IACUC) at Orient Bio Inc. (ORIENT-IACUC-17201).

### 2.2. Preparation of HemoHIM

HemoHIM was prepared according to the method described in our previous report [19]. In brief, three herbs used for antioxidant and immune response regulation in traditional Korean medicine, Angelica Radix (root of* Angelica gigas* Nakai), Cnidii Rhizoma (rhizome of* Cnidium officinale* Makino), and Paeonia Radix (root of* Paeonia japonica* Miyabe), were extracted for 4 h in boiling water to obtain a total extract of HIM-1. Half of the extract was precipitated by adding 4 volumes of 100% ethanol to obtain an ethanol-insoluble polysaccharide fraction. HemoHIM was prepared by adding the ethanol-insoluble polysaccharide fraction to the other half of HIM-1.

### 2.3. Cisplatin Injection and HemoHIM Administration

Cisplatin was dissolved in 0.5% carboxymethyl cellulose (CMC; Sigma, St. Louis, MO, USA) and mice were intraperitoneally injected with cisplatin 5 mg/kg b.w. on days 1, 6, and 11. HemoHIM was orally administered at doses of 100, 250, or 500 mg/kg b.w. daily on days 1-13. Mice were sacrificed and their blood was collected using ethylenediaminetetraacetic acid (EDTA) or heparin for complete blood count (CBC), NK cell activity assay, or flow cytometry. Organ weights of spleen and thymus were measured to calculate the organ index (%) according to the formula of (absolute organ weight)/(body weight at sacrifice)×100. The spleen was stored on ice in tubes for further isolation of splenocytes.

### 2.4. NK Cell Activity Analysis

The blood of all experimental groups was collected on day 14. Each 1 mL of blood was transferred to blood collection tube for assessing NK cell activity containing a patented stimulatory cytokine (Promoca™, ATGen, Sungnam, Republic of Korea). The collection tube was gently mixed within 30 min, the tube incubated for 20 h at 37°C. After incubation, each sample was centrifuged for 15 min. The supernatants were measured by the IFN-*γ* based assay using Murine NK activity kit (ATGen, Sungnam, Republic of Korea) according to the manufacturer's instructions.

### 2.5. Phagocytosis Assay

Peritoneal macrophages were isolated at 4 days after initial injection of 3% thioglycollate medium on day 14. Eight mL of ice-cold culture media (RPMI-1640 media, Invitrogen, Carlsbad, CA, USA) was injected into the peritoneal cavity, gently massaged, and then peritoneal fluid was collected. The collected fluid was transferred into tubes kept on ice. The cell suspensions were mixed with a lysing buffer at 37°C for 5 min to lyse RBCs. The macrophages were rinsed three times with RPMI-1640 media and then counted. The isolated macrophages were seeded in 96-well plates at a density of 5×10^4^ cells/well in 200 uL culture media. After 2 h, unattached cells were removed and the remaining cells were incubated for a day. The phagocytosis assay was performed using a CytoSelect 96-well phagocytosis assay kit (Cell Biolabs Inc., San Diego, CA, USA) according to the manufacturer's instructions.

### 2.6. Isolation of Splenocytes

Each excised spleen was pressed and sliced using two slide glasses and then placed onto a 40 *μ*m strainer. Filtrated cells were washed by RPMI-1640 media at 3,000 rpm for 5 min. The supernatant was removed and the cell pellets were treated with lysis buffer (BD PharmLyse Lysis buffer; BD Biosciences, San Diego, CA, USA) at 37°C for 5 min. The lysed cells were rinsed twice with RPMI-1640 media at 1,500 rpm for 5 min. The number of isolated cells was determined by counting with trypan blue stain (Thermo Fisher Scientific Inc., Waltham, MA, USA).

### 2.7. Flow Cytometric Analysis of CD4+ and CD8+T Lymphocytes

Collected blood (100 uL) was stained with 20 uL of PE-conjugated anti-mouse CD8 and FITC- conjugated anti-mouse CD4 antibodies (Biolegend, San Diego, CA, USA) for 1 hr in a dark room. Blood was stirred softly and incubated for 30 min in a cold dark room. Blood was mixed with 500 uL of OptiLyse® C no-wash lysing solution (Beckman Coulter Inc., Indianapolis, IN, USA) and incubated for 10 min in a cold dark room. The sample was centrifuged at 1,800 rpm for 5 min and the cell pellets were washed thrice using FACS buffer (PBS containing 0.05% NaN_3_, 5% FBS, pH 7.2) and centrifuged. The isolated pellet was added to 0.5 mL FACS buffer and was acquired on a BD FACS Aria™ III (BD Biosciences, Franklin Lakes, NJ, USA). Splenocyte (4x10^6^cells) was mixed with 200 uL FACS buffer (1XPBS, 5% FBS, 0.05% NaN_3_) and incubated for 30 min. The cells were stained with 20 uL of PE-Cy5 conjugated anti-mouse CD3, PE- conjugated anti-mouse CD8, and FITC- conjugated anti-mouse CD4 antibodies (Biolegend, San Diego, CA, USA) for 30 min in a cold dark room and then the cells were centrifuged at 1,500 rpm for 5 min. The cells were washed twice with FACS buffer, suspended in 250 uL PBS containing 1% formalin, and then acquired on a BD FACS Aria™ III.

### 2.8. Splenocyte Proliferation Assay

The splenocyte proliferation rate was measured by 3-[4,5-dimethylthiazol-2yl]-2,5-diphenyl-tetrazolium bromide (MTT) assay. Briefly, splenocytes were seeded in 96-well plates at 2.5×10^4^ cells/well in 100 uL RPMI-1640 media (10% FBS, and 1% Penicillin-Streptomycin) and treated with ConA (1 *μ*g/mL) for 2 days. After incubation, splenocyte proliferation was performed by TACS MTT cell proliferation assay kit (4890-25-K, Trevigen, Gaithersburg, MD, USA) according to the manufacturer's instructions.

### 2.9. Enzyme-Linked Immunosorbent Assay (ELISA)

Splenocytes were seeded on 24-well plates at 2.5×10^5^ cells/well with RPMI-1640 media (10% FBS, 1% penicillin-streptomycin) and stimulated with ConA (1 *μ*g/mL) for 2 days to measure cytokines. The media were centrifuged at 3,000 rpm for 5 min and then IL-2, IL-4, TNF-*α*, and IFN-*γ* were quantified in the supernatant by ELISA kits from MyBioSource (Atlanta, GA, USA) according to the manufacturer's instructions.

### 2.10. Statistical Analysis

Data were expressed as mean ± S.D., comparisons were made with One-Way Analysis of Variance (ANOVA) using SPSS statistics version 22 (IBM, Chicago, IL, USA), and* post hoc* testing was performed using Dunnett's test. Data with p value < 0.05 were considered statically significant.

## 3. Results

### 3.1. HemoHIM Restores Cisplatin-Induced Body Weight, Spleen and Thymus Weight Losses

**Figure 1:**
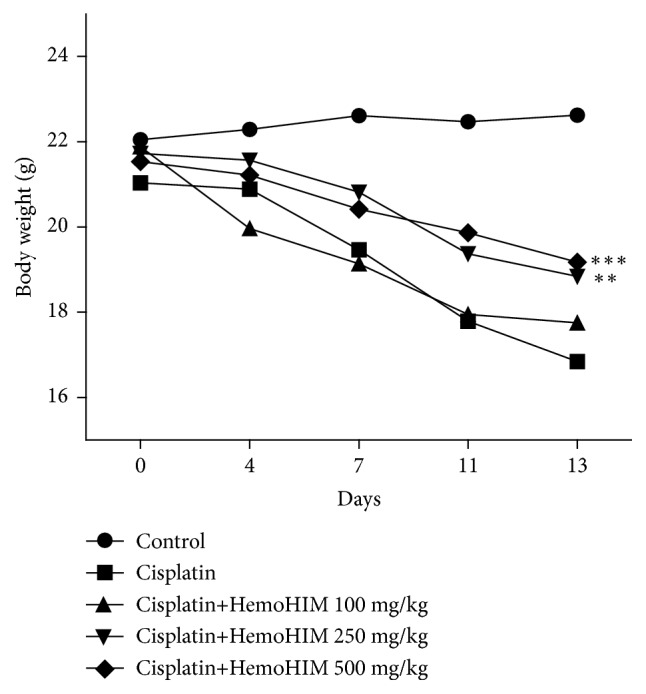
Effect of HemoHIM on the body weight in cisplatin-treated mice. The body weight was measured on days 0, 4, 7, 11, and 13. Data are expressed as mean ± SD (n=7). Comparison was made between cisplatin and HemoHIM groups. Significant difference from cisplatin group (*∗∗p<0.01*, *∗∗∗p<0.001*).

Body weight significantly decreased after cisplatin treatment and did not recover until day 13 after treatment (Figure 1). However, HemoHIM treatment (250 and 500 mg/kg) inhibited the body weight loss in cisplatin-treated mice.  Table 1 shows the relative organ weights calculated as “organ weight/final body weight x 100”. The relative weight of thymus was decreased by cisplatin treatment (*p<0.001*).

### 3.2. HemoHIM Enhances NK Cell Activity in Cisplatin-Treated Mice

**Figure 2:**
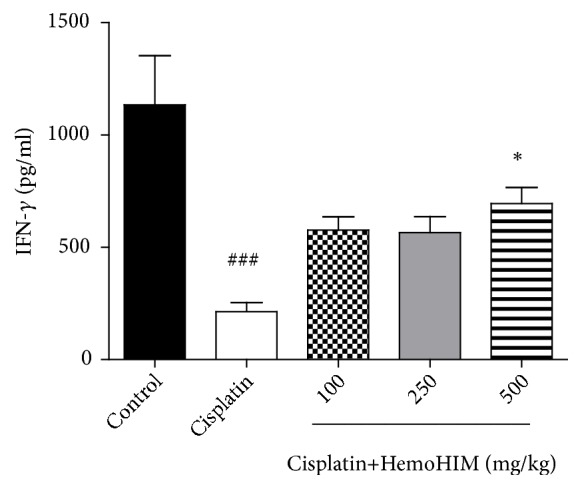
Effect of HemoHIM on NK cell activity in cisplatin-treated mice. NK cell activity was measured using heparin-collected blood on day 14. Significant difference from control (###* p<0.001*) and from cisplatin group (*∗p<0.05*).

NK cells act through direct cytotoxic attacks on their targets or through the ability to secrete cytokines and chemokines. It was previously reported that NK cell activity was elevated by HemoHIM treatment [11, 20] and the same trend was observable in this result. As shown in Figure 2, NK cell activity was decreased in cisplatin group (213.054±41.912 ng/mL) compared to control group (1134.295±218.421 ng/mL) (*p<0.001*). However, the suppression of NK cell activity by cisplatin was significantly alleviated by treatment with HemoHIM (HemoHIM 100 mg/kg; 576.275±60.128 ng/mL, HemoHIM 250 mg/kg; 565.593±71.272 ng/mL, HemoHIM 500 mg/kg; 695.176±71.270 ng/mL,* p<0.05*).

### 3.3. HemoHIM Protects the Phagocytosis Activity of Macrophages in Cisplatin-Treated Mice

**Figure 3:**
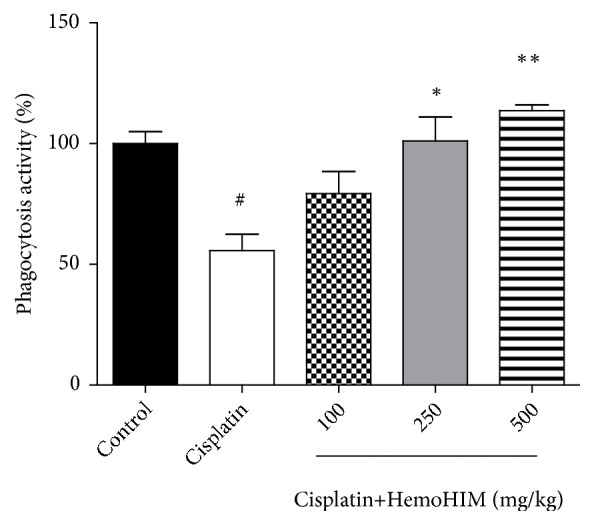
Effect of HemoHIM on phagocytosis activity of macrophage in cisplatin-treated mice. The phagocytosis assay was measured using phagocytosis assay kit. Significant difference from control (#* p<0.05*) and from cisplatin group (*∗p<0.05*, *∗∗p<0.01*).

Macrophage phagocytosis initiates the innate immune response, which turns on the adaptive response [21]. The phagocytotic activity of macrophages was decreased by cisplatin treatment (55.756±6.706%) compared to control group (100±4.922%). HemoHIM treatment dose-dependently protected macrophage phagocytotic activity from cisplatin toxicity (HemoHIM 100 mg/kg; 79.287±9.226%, HemoHIM 250 mg/kg; 101.038±9.931%,* p<0.05*, HemoHIM 500 mg/kg; 113.645±2.427 %,* p<0.01*) (Figure 3).

### 3.4. HemoHIM Increases CD4+ T Lymphocytes in Cisplatin-Treated Mice

**Figure 4:**
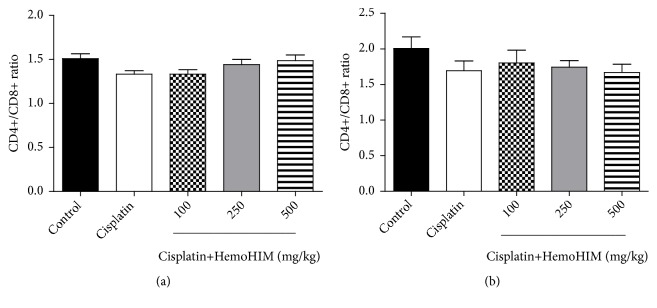
Effect of HemoHIM on the ratio of CD4+/CD8+ T cell in cisplatin-treated mice. The blood was stained with PE-conjugated anti-mouse CD8 and FITC-conjugated anti-mouse CD4. The splenocytes were stained with PE-Cy5 conjugated anti-mouse CD3, PE-conjugated anti-mouse CD8, and FITC-conjugated anti-mouse CD4. The stained cells were acquired on FACScan. (a) The ratio of CD4+/CD8+ in splenocytes and (b) the ratio of CD4+/CD8+ in blood.

The CD4+^/^CD8+ ratio indicates the ratio of helper T cells to cytotoxic T cells. A declining CD4+/CD8+ ratio denotes a lack of resistance to infection such as HIV infection, immunodeficiency, and autoimmunity [22–24]. Thus, we investigated the ratio of CD4+/CD8+ in both splenocytes and blood (Figure 4). The ratio of CD4+/CD8+ was not significantly different in between cisplatin treatment and HemoHIM treatment in both blood and splenocyte. However, CD4+ T lymphocytes tended to decrease in cisplatin group compared to control group (Table 2). The decreased CD4+ T lymphocytes were significantly alleviated by HemoHIM treatment at 250 and 500 mg/kg in splenocytes (*p<0.05, p<0.01*).

### 3.5. HemoHIM Increases Cell Proliferation and Regulates the Secretion of Cytokines in Splenocytes Obtained in Cisplatin-Treated Mice

**Figure 5:**
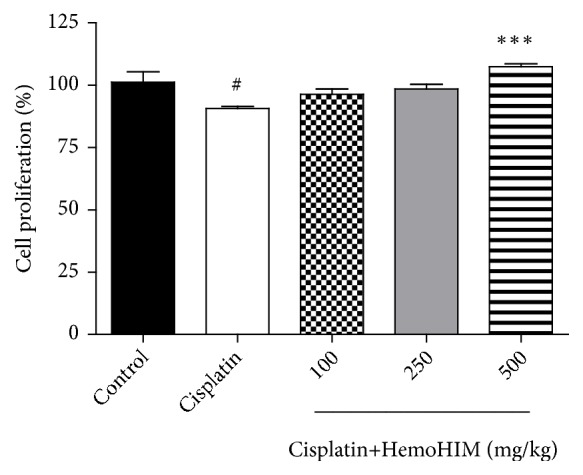
Effect of HemoHIM on cell proliferation of splenocytes in cisplatin-treated mice. The splenocyte proliferation rate was measured by MTT assay. Significant difference from control (#* p<0.05*) and significant difference from cisplatin-treated group (*∗∗∗p<0.001*).

**Figure 6:**
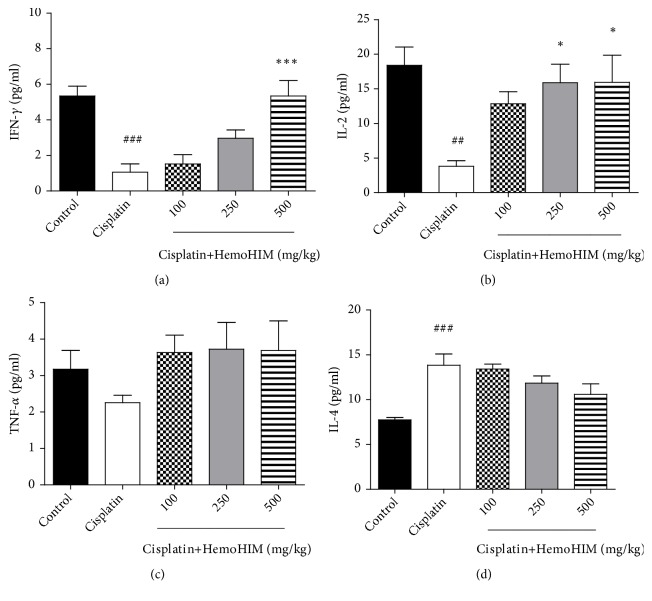
Effect of HemoHIM on the secretion of IFN-*γ*, IL-2, TNF-*α*, and IL-4 in splenocytes obtained in cisplatin-treated mice. The splenocytes were treated with ConA and the cytokines released into media were measured using ELISA kits. (a) IFN-*γ* secretion, (b) IL-2 secretion, (c) TNF-*α* secretion, and (d) IL-4 secretion. Significant difference from control (##*p<0.01*, ###*p<0.001*) and from cisplatin group (*∗p<0.05*, *∗∗∗p<0.001*).

We investigated the effects of HemoHIM on the proliferation of splenocytes, by isolating and culturing spleen tissue with ConA. The cisplatin group showed reduced proliferation compared to the control group (Figure 5), consistent with previous studies done with immunosuppressive drugs that inhibited mitogen-induced lymphocyte proliferation [25]. However, HemoHIM treatment restored the splenocyte proliferation to the control level (HemoHIM 500 mg/kg,* p<0.001*). Further, we investigated whether HemoHIM would regulate the secretion of Th1- and Th2-associated cytokines in splenocytes. Cisplatin reduced Th1-associated cytokines (IL-2 and IFN-*γ*) while HemoHIM treatment dose-dependently restored the production of IL-2 and IFN-*γ*. The production of TNF-*α* showed similar trends though it was not statistically significant. Cisplatin increased the production of Th2-associated cytokine (IL-4), while HemoHIM treatment showed a trend to reduce secretion of IL-4 (Figure 6).

### 3.6. Figures, Tables, and Schemes

See Figures 1–6 and Tables 1 and 2.

## 4. Discussion

Several natural products have been studied to identify new immunomodulators. Ginseng has the effect of immunomodulatory enhancing the expression of IL-1 and TNF [26] and* Curcuma longa* inhibit Th1/Th2 cytokine imbalance and proinflammatory cytokine production [27]. The immune regulation of polysaccharides isolated from natural extracts has also been reported [28, 29]. HemoHIM is a herbal medicine composed of hot water extracts of* Angelica gigas* Nakai,* Cnidium officinale* Makino, and* Paeonia japonica* Miyabe with enhanced crude polysaccharide content, which are active ingredients that enhance immunity and hematopoiesis. HemoHIM has anticancer effect while reducing chemotherapy side effect [30], aids the recovery from immune imbalance [20], and shows anti-inflammatory effects in respiratory inflammation [31]. Although there have been studies that investigated the effects of HemoHIM in inflammatory diseases and immunity, there have been no studies in the context of immunosuppression by cisplatin, which is used as an immunosuppressive agent and an anticancer agent. Therefore, the present study investigated the immunomodulatory effect of HemoHIM on cisplatin-induced immune-suppressed model.

Cisplatin is a treatment for several types of cancer having side effects on the respiratory, nervous, and vascular systems [32]. In particular, bone-marrow inhibition is known to cause leukopenia, anemia, and hematologic toxicity as side effects of cisplatin [33]. Cisplatin also acts as a representative redox cycler, causing direct damage to various organs and resulting in significant weight loss [34]. In the present study, mice administered with cisplatin showed adverse effects on body weight, similar to the findings of Park et al. [14] and Shruthi et al. [17]. However, HemoHIM treatment groups increased the body weight, which is comparable with cisplatin-treated group. The relative weight of thymus in cisplatin treatment group only decreased compared to control group. However, HemoHIM showed no significant effects on spleen and thymus weights.

It has been reported that NK cell activity is decreased in some patients such as those with stomach cancer, breast cancer, or prostate cancer, indicating weakening of the immune system [35, 36]. In the present study, NK cell activity was assessed by measuring IFN-*γ* and was found to be significantly increased in HemoHIM-treated mice compared to cisplatin-treated mice. These findings are consistent with previous study in which HemoHIM treatment resulted in increased NK cell activity in splenocytes [20]. In addition, the phagocytic activity of macrophages increased in a dose-dependent manner in HemoHIM-treated mice.

T lymphocytes constitute a heterogeneous cell population with two major phenotypes, namely, CD4 or CD8 markers on the surface. CD4+ T lymphocytes are usually associated with helper/inducer functions while CD8+ T lymphocytes are generally associated with cytotoxic/suppressor activity [22]. The ratio of CD4+/CD8+ and absolute number of CD4+ determine whether the immune system is strong and predict the risks of complications and infections. In addition, it is useful to periodically compare the number of CD4 cells with other lymphocytes because CD4 cells are generally destroyed faster than other lymphocytes. The reduction in the absolute number of CD4+ provides a basis for determining the diagnosis of immune deficiency syndrome and the monitoring of disease progression and the direction of treatment [37, 38]. In particular, the CD4+ level is used as a criterion for determining disease state and treatment policy in HIV patients [39]. In our study, the ratio of CD4+/CD8+ was not significantly different between the cisplatin-treated group and HemoHIM-treated group. However, reduced CD4+ T lymphocyte ratio by cisplatin in splenocytes was significantly restored by HemoHIM treatment similar to the findings of Lee et al. [40]. Previous studies showed that immunosuppressive reagents inhibited the proliferation of lymphocytes and splenocytes [41, 42]. The present study showed that cisplatin inhibited splenocyte proliferation, but HemoHIM was restored in a concentration-dependent manner. The cisplatin-induced alteration of splenocyte cytokine secretion was alleviated by HemoHIM treatment. The levels of Th-1 cytokines including IL-2, IFN-*γ*, and TNF-*α* were higher and the level of Th-2 cytokine, IL-4, was lower compared to cisplatin-treated group. These results demonstrated that HemoHIM modulates the Th1/Th2-mediated immune response in cisplatin-induced immune-suppressed mice.

## 5. Conclusion

The herbal preparation, HemoHIM, significantly inhibited cisplatin-induced immunosuppression, through promoting NK cell activity, the phagocytosis activity of macrophages, proliferation of splenocytes, and Th1-related cytokine production. Therefore, HemoHIM can be a potent immunomodulatory agent overcoming toxicity and side effects.

## Figures and Tables

**Table 1:** Effect of HemoHIM on spleen and thymus weight change in cisplatin-treated mice.

Group	Relative organ weight (%)*∗*	
	Spleen	Thymus
Control	0.218 ± 0.013	0.171 ± 0.013
Cisplatin	0.193 ± 0.018	0.049 ± 0.024 ^###^
Cisplatin + HemoHIM 100 mg/kg	0.205 ± 0.015	0.046 ± 0.037
Cisplatin + HemoHIM 250 mg/kg	0.212 ± 0.022	0.053 ± 0.032
Cisplatin + HemoHIM 500 mg/kg	0.197 ± 0.021	0.047 ± 0.033

The body weight was measured on days 0, 4, 7, 11, and 13 and organ weight was measured on day 14.

*∗*Data are expressed as mean ± SD (n=7). Relative organ weight (%) = (organ weight)/(final body weight) x 100.

Comparison was made between control and cisplatin groups (###*p<0.001*).

**Table 2:** Effect of HemoHIM on flow cytometric analysis CD4+ and CD8+T lymphocytes in cisplatin-treated mice.

Group	Splenocyte		Whole blood	
	CD4^+^(%)	CD8^+^(%)	CD4^+^(%)	CD8^+^(%)
Control	54.77 ± 1.86	36.46 ± 2.66	15.04 ± 2.69	7.84 ± 2.32
Cisplatin	51.91 ± 1.28	39.20 ± 2.46	14.87 ± 2.76	8.87 ± 1.07
Cisplatin + HemoHIM 100 mg/kg	53.47 ± 2.39	40.36 ± 2.57	15.27 ± 2.77	8.70 ± 1.54
Cisplatin + HemoHIM 250 mg/kg	56.07 ± 2.37^*∗*^	39.13 ± 2.49	15.63± 1.92	9.09 ± 1.57
Cisplatin + HemoHIM 500 mg/kg	56.73 ± 3.20^*∗∗*^	38.41 ± 2.39	18.94 ± 3.52	11.57 ± 2.62

Significant difference from cisplatin group (*∗p<0.05*, *∗∗p<0.01*).

## Data Availability

The data used to support the findings of this study are available from the corresponding author upon request.
